# The Rare *IL22RA2* Signal Peptide Coding Variant rs28385692 Decreases Secretion of IL-22BP Isoform-1, -2 and -3 and Is Associated with Risk for Multiple Sclerosis

**DOI:** 10.3390/cells9010175

**Published:** 2020-01-10

**Authors:** Paloma Gómez-Fernández, Aitzkoa Lopez de Lapuente Portilla, Ianire Astobiza, Jorge Mena, Andoni Urtasun, Vivian Altmann, Fuencisla Matesanz, David Otaegui, Elena Urcelay, Alfredo Antigüedad, Sunny Malhotra, Xavier Montalban, Tamara Castillo-Triviño, Laura Espino-Paisán, Orhan Aktas, Mathias Buttmann, Andrew Chan, Bertrand Fontaine, Pierre-Antoine Gourraud, Michael Hecker, Sabine Hoffjan, Christian Kubisch, Tania Kümpfel, Felix Luessi, Uwe K. Zettl, Frauke Zipp, Iraide Alloza, Manuel Comabella, Christina M. Lill, Koen Vandenbroeck

**Affiliations:** 1 Neurogenomiks Laboratory, University of the Basque Country (UPV/EHU), 48940 Leioa, Spain; paloma.gomez.fernandez@gmail.com (P.G.-F.); aitzkoa.lopez_de_lapuente_portilla@med.lu.se (A.L.d.L.P.); nireaspe@hotmail.com (I.A.); jorgemena65@outlook.com (J.M.); a.urtasun.arricaberri@gmail.com (A.U.); iraide.alloza@gmail.com (I.A.); 2 Department of Laboratory Medicine, Lund University, SE-221 00 Lund, Sweden; 3 Inflammation & Biomarkers Group, Biocruces Bizkaia Health Research Institute, 48903 Barakaldo, Spain; 4 Genetic and Molecular Epidemiology Group, Lübeck Platform for Genome Analytics, Institutes of Neurogenetics and Cardiogenetics, University of Lübeck, 23552 Lübeck, Germany; vivian.altmann@uni-luebeck.de (V.A.); christina.lill@uni-luebeck.de (C.M.L.); 5 Department of Cell Biology and Immunology, Instituto de Parasitología y Biomedicina López Neyra (IPBLN), CSIC, 18002 Granada, Spain; lindo@ipb.csic.es; 6 Multiple Sclerosis Group, Biodonostia Research Institute, Paseo Doctor Begiristain, s/n, 20014 San Sebastián, Spain; David.otaegui@biodonostia.org (D.O.); tamara.castillo@biodonostia.org (T.C.-T.); 7 Instituto de Investigación Sanitaria del Hospital Clínico San Carlos, IdISSC, 28014 Madrid, Spain; elena.urcelay@salud.madrid.org (E.U.); lauraep80@yahoo.es (L.E.-P.); 8 Department of Neurology, Cruces Hospital, S/N, 48903 Barakaldo, Spain; alfredo.r.antiguedad.z@gmail.com; 9 Servei de Neurologia-Neuroimmunologia, Centre d’Esclerosi Múltiple de Catalunya (Cemcat), Institut de Recerca Vall d’Hebron (VHIR), Hospital Universitari Vall d’Hebron, Universitat Autònoma de Barcelona, 08007 Barcelona, Spain; sunnymalhotra4u24@gmail.com (S.M.); xavier.montalban@cem-cat.org (X.M.); manuel.comabella@vhir.org (M.C.); 10 Department of Neurology, Medical Faculty, Heinrich-Heine University Düsseldorf, 40225 Düsseldorf, Germany; Orhan.Aktas@uni-duesseldorf.de; 11 Department of Neurology, University of Wuerzburg, 97080 Wuerzburg, Germany; Mathias.Buttmann@ckbm.de; 12 Department of Neurology, Caritas Hospital, 97980 Bad Mergentheim, Germany; 13 Department of Neurology, Inselspital Bern, Bern University Hospital, University of Bern, 3011 Bern, Switzerland; andrew.chan@insel.ch; 14 INSERM, Sorbonne University, Assistance Publique-Hopitaux de Paris (AP-HP), UMR 974 and Neuro-Myology Service, University Hospital Pitié-Salpêtrière, 75013 Paris, France; bertrand.fontaine@sorbonne-universite.fr; 15 Nantes Université, CHU, INSERM, Centre de Recherche en Transplantation et Immunologie, UMR 1064, ATIP-Avenir, Equipe 5, 44093 Nantes, France; pierre-antoine.gourraud@univ-nantes.fr; 16 CHU de Nantes, INSERM, CIC 1413, Pôle Hospitalo-Universitaire 11: Santé Publique, Clinique des données, 44000 Nantes, France; 17 Department of Neurology, Neuroimmunological Section, University of Rostock, 18147 Rostock, Germany; michael.hecker@rocketmail.com (M.H.); uwe.zettl@med.uni-rostock.de (U.K.Z.); 18 Department of Human Genetics, Ruhr-University Bochum, 44801 Bochum, Germany; sabine.hoffjan@rub.de; 19 Institute of Human Genetics, University Medical Center Hamburg-Eppendorf, 20246 Hamburg, Germany; c.kubisch@uke.de; 20 Institute of Clinical Neuroimmunology, Ludwig-Maximilians University, 80333 Munich, Germany; tania.kuempfel@med.uni-muenchen.de; 21 Department of Neurology, Focus Program Translational Neuroscience, University Medical Center of the Johannes Gutenberg University Mainz, 55116 Mainz, Germany; luessi@uni-mainz.de (F.L.); frauke.zipp@unimedizin-mainz.de (F.Z.); 22 Section for Translational Surgical Oncology and Biobanking, Department of Surgery, University of Lübeck and University Medical Center Schleswig-Holstein, Campus Lübeck, 23552 Lübeck, Germany; 23 Ageing Epidemiology Research Unit, School of Public Health, Imperial College, London SW71, UK; 24 Ikerbasque, Basque Foundation for Science, 48013 Bilbao, Spain

**Keywords:** *IL22RA2*, IL-22 binding protein isoform, mutation, signal peptide, multiple sclerosis, autoimmune

## Abstract

The *IL22RA2* locus is associated with risk for multiple sclerosis (MS) but causative variants are yet to be determined. In a single nucleotide polymorphism (SNP) screen of this locus in a Basque population, rs28385692, a rare coding variant substituting Leu for Pro at position 16 emerged significantly (*p* = 0.02). This variant is located in the signal peptide (SP) shared by the three secreted protein isoforms produced by *IL22RA2* (IL-22 binding protein-1(IL-22BPi1), IL-22BPi2 and IL-22BPi3). Genotyping was extended to a Europe-wide case-control dataset and yielded high significance in the full dataset (*p* = 3.17 × 10^−4^). Importantly, logistic regression analyses conditioning on the main known MS-associated SNP at this locus, rs17066096, revealed that this association was independent from the primary association signal in the full case-control dataset. In silico analysis predicted both disruption of the alpha helix of the H-region of the SP and decreased hydrophobicity of this region, ultimately affecting the SP cleavage site. We tested the effect of the p.Leu16Pro variant on the secretion of IL-22BPi1, IL-22BPi2 and IL-22BPi3 and observed that the Pro16 risk allele significantly lowers secretion levels of each of the isoforms to around 50%–60% in comparison to the Leu16 reference allele. Thus, our study suggests that genetically coded decreased levels of IL-22BP isoforms are associated with augmented risk for MS.

## 1. Introduction

Multiple sclerosis (MS) is a chronic inflammatory CNS disorder triggered by environmental factors in individuals with a susceptible genetic background. Current research implies more than 230 autosomal risk variants, many of which are located within or close to genes exerting functions in the peripheral immune system or in CNS-resident microglia [1]. Single nucleotide polymorphisms (SNPs) located at the interleukin-22 receptor subunit alpha-2 (*IL22RA2*) gene locus were originally reported to be associated with risk for MS in Scandinavian [2] and Basque [3] populations. A series of genome-wide association studies (GWAS)-based approaches have subsequently firmly established the association of *IL22RA2* with risk for MS [1,4,5,6].

The main known function of *IL22RA2* is to produce interleukin-22 binding protein (IL-22BP), a secreted inhibitor of IL-22. IL-22, a member of the IL-10 family, is produced by a wide range of immune cells and can exert both pro- and anti-inflammatory effects [7,8]. Various lines of evidence suggest that the IL-22/IL-22BP axis has an important function in MS and neuroinflammation. *Il22ra2*-deficient mice experience a more benign course of disease in the experimental autoimmune encephalomyelitis (EAE) model [9]. The IL-22 receptor is highly expressed in blood–brain barrier (BBB) endothelial cells from patients with MS but not in healthy controls, and IL-22 contributes to the permeabilization of the BBB and recruitment of CD4^+^ T lymphocytes to the CNS [10]. A decrease of IL-22 levels correlates with the recovery phase of EAE in rats [11], and serum levels of IL-22 were found to be elevated in MS patients compared to healthy controls [12]. Perriard et al. [13] demonstrated that IL-22 targets astrocytes in the human brain and confers increased survival to these cells. They also found higher expression of IL-22BP mRNA in monocytes and monocyte-derived dendritic cells of MS patients compared to healthy controls.

*IL22RA2* is capable of expressing three partially distinct isoforms that share an identical signal peptide (SP) at their N-terminus and lack intracellular and transmembrane domains but differ in their binding capacity of IL-22. Isoform 2 (UniProt nomenclature) shows the highest capacity of binding and inhibiting IL-22 [14,15], with a 20- to 1000-fold higher affinity than a soluble variant of the signal-transducing cell surface receptor [16,17,18]. Isoform 3 has also been demonstrated to bind IL-22, although with a similar affinity to that of the cell surface receptor [16,19]. Recently, we showed that the longest isoform, i.e., isoform 1, is not capable of binding IL-22 and displays hallmarks of a poorly secreted, intracellularly retained protein with intrinsic capacity to trigger the unfolded protein response (UPR; [20]).

Although the association of *IL22RA2* with MS is now established and accumulating evidence points to an influence of IL-22 and *IL-22RA2* in EAE and MS, the mechanism underlying the genetic association remains elusive. Here, we performed a SNP screen of the *IL22RA2* locus in a Basque population in order to localize the most important association signal(s) within this locus and confirmed association of an infrequent coding SNP in a European cohort. We used dedicated in silico and wet experimentation methods to discover potentially causal variants that may explain the association of this gene with MS.

## 2. Materials and Methods

### 2.1. Patients and Controls

All patients were diagnosed with definite MS [21,22]. Written informed consent was obtained from all subjects, and the study was approved by the local ethics committees. Table 1 shows the clinical and demographic data of the patients and controls enrolled in this study. The fine-mapping was completed in the Bilbao dataset, comprising patients registered at the Basurto hospital (Bilbao, Basque Country, Spain) and controls provided by the Basque BioBank for Research-OEHUN (www.biobancovasco.org). Additionally, genotyping data of three SNPs (rs276466, rs10484798 and rs6570136) in the Bilbao cohort were available from the aforementioned screening [3], and these were included in the haplotype and logistic regression analyses.

The non-synonymous SNP, rs28385692, was further genotyped in additional patients and age-, gender- and ethnicity-matched controls from Donostia (Instituto Biodonostia, Basque Country, Spain), Barcelona (Hospital Vall d’Hebron), Madrid (Hospital Clínico S. Carlos), Andalucía (Instituto de Parasitología y Biomedicina “Lopez-Neyra”), Germany (various centers) and France (various centers) (Table 1). Considering the SNP with the lowest MAF (rs28385692, 1000G European frequency = 0.032) and combining all datasets, this study had over 80% statistical power to detect allelic odds ratios ≥ 1.18 with a type-1-error rate of alpha = 0.05 (calculated with the Genetic Power Calculator allelic test [23]). When considering only the Bilbao dataset, we had over 80% power to detect odds ratios ≥ 1.8, assuming a MAF = 0.032 and a type-1-error of alpha = 0.05.

The lead SNP from the 2011 GWAS [6], rs17066096, was also genotyped in all the above-mentioned validation cohorts, excluding France (Table 1). Additionally, data on the most significantly associated SNP in the fine mapping, rs202573, were available from Andalucía, Barcelona and Madrid from our previous study [3], and this SNP was newly genotyped in the Donostia and Germany cohorts.

### 2.2. Selection and Genotyping of SNPs

Ten haplotype-tagging SNPs were selected based on genotype data from the CEU + TSI population (HapMap release #27) using an *r*^2^ cut-off = 0.9 and a MAF = 0.1 (with the Multimarker Tagger Algorithm available on the HapMap website, www.hapmap.org, now defunct). In addition, SNP rs13217897 was selected based on its association with MS as reported by an independent group [2], and rs28385692 was included because of its potential functional effect, given its location in the coding region. Haplotype-tagging SNPs in the Bilbao cohort and rs28385692 in the Donostia cohort were genotyped using the iPLEX Sequenom MassARRAY platform in the Spanish National Genotyping Center (CEGEN, Santiago de Compostela, Spain, www.cegen.es). Genotyping of rs17066096, rs202573 and rs28385692 in the validation cohorts was performed using Taqman^®^ Genotyping Assays, following the manufacturer’s instructions. The genotyping efficiency was above 95% in all datasets, and all SNPs were in Hardy–Weinberg equilibrium in controls in each dataset.

### 2.3. Statistical Analysis

The statistical analysis was performed using PLINK v1.07 (http://zzz.bwh.harvard.edu/plink/) [24]. For the association analysis, a logistic regression with an additive model, adjusted for sex, was applied. The independence of association signals was assessed using a conditional logistic regression analysis. A chi-squared test was used to check for the Hardy–Weinberg equilibrium. A haplotype analysis was performed using Haploview v.4.2 (https://www.broadinstitute.org/haploview/haploview) [25]. Only the samples that had genotyping data available for all the SNPs were used for the haplotype calculation (for the Bilbao cohort, 375 cases and 441 controls; for the joint datasets, 6545 cases and 5713 controls). Haplotype blocks were defined by the confidence interval method [26]. Permutations were applied (*n* permutations = 1000) to correct for multiple comparisons in the haplotype analysis. Statistical power was calculated using the CaTS power calculator at www.sph.umich.edu/csg/abecasis/CaTS/ [27]. Secretion levels of Leu16 IL-22BP isoforms compared to those of Pro16 variants were compared used Student’s unpaired *t*-test.

### 2.4. Functional Annotation of SNPs

Each of the associated SNPs and their proxies (*r*^2^ ≥ 0.8 in European populations of the 1000 Genomes pilot project as retrieved from the SNAP Proxy Search tool (https://www.broadinstitute.org/mpg/snap/) [28] were functionally annotated using the Ensembl Variant Effect Predictor (VEP) (https://www.ensembl.org/info/docs/tools/vep/index.html) [29] and the RegulomeDB (http://www.regulomedb.org/) [30].

### 2.5. In silico Analysis of the Effect of the Leu to Pro Transition Coded by rs28385692 on Signal Peptide Characteristics

PredictSNP web server (https://loschmidt.chemi.muni.cz/predictsnp1/) [31], Meta-SNP (http://snps.biofold.org/meta-snp/) [32] and Ensembl VEP tool (http://www.ensembl.org/Homo_sapiens/Variation/Mappings?db=core;r=6:137161203-137162203;v=rs28385692;vdb=variation;vf=104559210#ENST00000296980_104559210_G_tablePanel) [29,33] were used to estimate the functional effect of p.Leu16Pro mutation on IL-22BP. The SignalP 3.0 [34], Phobius [35], PsiPred [36], SignalP 5.0 (http://www.cbs.dtu.dk/services/SignalP/) [37], PrediSi [38], and Signal-3L tools were used to predict the effects of the p.Leu16Pro substitution on SP function in IL-22BP. Secondary structure features of the wildtype/mutant proteins were predicted by PsiPred [39], RaptorX (http://raptorx.uchicago.edu/) [40] and SABLE [41].

### 2.6. p.Leu16Pro Mutagenesis of the Three IL-22BP Isoforms

The expression plasmid for *IL22RA2v1* was constructed as described in our previous work [20], *IL22RA2v2* and *IL22RA2v3* expression plasmids were purchased from OriGene Technologies (RC219095, Rockville, MD, USA) and GenScript (Ohu00490, Piscataway, NJ, USA), respectively. The p.Leu16Pro mutants of IL-22BPi1, 2 and 3 were generated using the GENEART^®^ site-directed mutagenesis system (A13282, Invitrogen, Waltham, MA, USA) from the *IL22RA2v1*, *2* and *3* expression plasmids following the manufacturer’s instructions. The site-directed mutagenesis primer design was also done following the manufacturer’s instructions. Briefly, both primers contained the desired mutation centrally located and were 100% complementary with no overhangs, and with lengths between 30 and 45 nucleotides. The designed primers, purchased from IDT, were purified by HPLC to increase mutagenesis efficiency. PCR was performed using a Verity thermocycler (Applied Biosystems, Waltham, MA, USA) with the following primers: IL22RA2_p.Leu16Pro_FW: 5′-TCATCAGTTTCTTCCCTACTGGTGTAGCAGG-3′ and IL22RA2_p.Leu16Pro _RV: 5′-CCTGCTACACCAGTAGGGAAGAAACTGATGA-3′. The PCR conditions used were: 1 cycle at 37 °C for 20 min and 94 °C for 45 s, 18 cycles at 94 °C for 45 s, 57 °C for 45 s and 72 °C for 6 min, 1 cycle at 72 °C for 10 min and a final holding stage at 4 °C. The resulting constructs were sequenced to confirm point mutations of *IL22RA2* variant constructs. The p.Leu16Pro mutant plasmids were transformed into DH5α-T1R *E. coli* competent cells following the manufacturer’s instructions. Plasmids were purified with EndoFree Plasmid Kit (Qiagen, Hilden, Germany), quantified by a NanoDrop Spectrophotometer (ThermoFisher Scientific, Waltham, MA, USA) and visually assessed via agarose gel electrophoresis.

### 2.7. Assessment of Effect of p.Leu16Pro Variant on Secretion of IL-22BP Isoforms

HEK293 cells were cultured in Dulbecco’s Modified Eagle Medium (DMEM: D5796, Sigma, St. Louis, MO, USA) supplemented with 10% fetal bovine serum (FBS; F9665, Sigma, St. Louis, MO, USA) at 37 °C and 5% CO_2_ in a humidified incubator. Cells were seeded on a 24-well plate at 500 μL/well at a density of 3 × 10^4^ cells/well. Cells were transfected with the indicated expression plasmids when they reached 60%–70% confluency using MACSfectin Reagent (130-098-412, Miltenyi, Bergisch Gladbach, Germany). Twenty-four hours after transfection, conditioned media were collected and cells washed three times with cold PBS prior cell lysis in RIPA buffer (25 mM Tris-HCl, 150 mM NaCl, pH 7.6; plus 1% N-40, 1% sodium deoxycholate, and 0.1% SDS) in the presence of protease inhibitors (11697498001, Roche, Basel, Switzerland). All cell lysates were quantified for total protein using BCA kit (23225, ThermoFisher Scientific); equal amounts of total protein were resuspended in reducing loading buffer and resolved on SDS-PAGE for further immunoblotting. For acetone precipitation of conditioned media, 4 h prior media collection, cells were carefully washed five times with pre-warmed serum-free medium (SFM; 12-764Q, Lonza, Basel, Switzerland) to remove serum proteins and SFM supplemented with L-glutamine (G5792, Sigma) was added for a further 4 h. Conditioned SFM were acetone precipitated with four volumes of ice-cold acetone and incubated on ice for 10 min followed by centrifugation at 21,000× *g* a 4 °C. Pellets were resuspended in reducing loading buffer and resolved on SDS-PAGE for further immunoblotting. The antibodies used in this study are the following: anti-FLAG (1:1000; 2043-1-AP, Proteintech, Rosemont, IL, USA); anti-IL-22BP (1:1000; AF1087 and BAF1087, R&D Systems, Minneapolis, MN, USA); anti-IL-22BP (1:1000; ab133965, Abcam, San Francisco, CA, USA); anti-tubulin (1:1000; A01490, GenScript, Piscataway, NJ, USA) and all HRP-conjugated secondary antibodies were purchased from Jackson ImmunoResearch. IL-22BP ELISA capable of detecting the three isoforms was performed as previously described [20].

### 2.8. Replacement of the SP of IL-22BPi2 with the IL17A SP

The IL17SP_IL22RA2v2 construct was generated from two overlapping fragments; the first one containing the terminal *SgfI* restriction site followed by the sequence of the SP of IL-17A and the beginning of IL-22BPi2 mature protein, and the second one, containing the end of IL-17A SP followed by IL-22BPi2 mature protein flanked by *Mlu* restriction site. The overlapping fragment was amplified and digested with *SgfI* and *MluI* restriction enzymes and cloned into pCMV6-Entry vector. Primers for amplification of the IL17A_Signal peptide fragment were: FW: 5′-GCCGCGATCGCCATGACTCCTGGGAAGACC-3′ and RV:5′-AGGCTTCA GAGACTCATGCGTTGACTGAGTGATTGTGATTCCTGCCTTCACTATGGCCTCCAGGCTC-3′. Primers for amplification of the *IL22RA2v2* mature fragment were: FW 5′-GAGCCTGGAGGCCATAGTGAAGGCAGGAATCACAATCACTCAGTCAACGCATGAGTCTCTG-3′ and RV: 5′-CGTACGCGTTGGAATTTCCACACATCTCTC-3′.

### 2.9. Flow Cytometry Analysis

HEK293 cells were transfected with expression vectors for wild-type or p.Leu16Pro mutant IL-22BPi2, collected after 24 h and washed with flow cytometry buffer (FC Buffer; PBS, 0.5% BSA and 2mM EDTA, pH 7.2). Single cell suspensions were fixed with 4% paraformaldehyde for 10 min at room temperature followed by permeabilization with 90% of ice-cold methanol for 30 min at 4 °C. Cells were blocked with 1% BSA for 15 min at RT, incubated with anti-IL-22BP primary antibody (1:250; 66190, Proteintech) for 30 min at room temperature and followed by another 30-minute incubation period with anti-mouse-FITC conjugated secondary antibody (1:500; AMI4608, ThermoFisher) protected from light. Two washes were performed after each step with FC buffer. Immunostained cells were analyzed using a MACSQuant Analyzer (Miltenyi).

## 3. Results

### 3.1. The p.Leu16Pro coding SNP rs28385692 is Associated with Risk for Multiple Sclerosis

**Figure 1:**
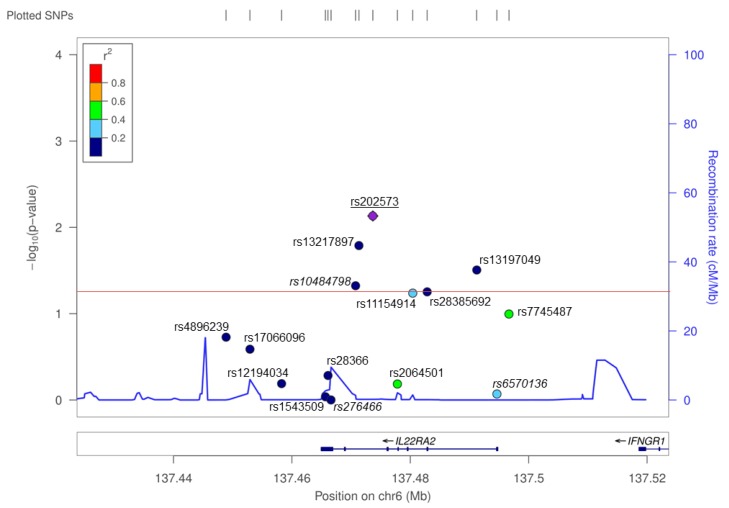
A single nucleotide polymorphism (SNP) screen of the *IL22RA2* locus in the Bilbao dataset. SNPs, depicted with dots in different colors depending on *r*^2^ values with respect to the index SNP rs202573, are plotted as a function of their log-converted *p*-value (left Y axis) and their position on chromosome 6 according to hg19 assembly of the human genome (X axis). The recombination rate across the locus is provided on the right Y axis. The red line represents the significance threshold (*p* = 0.05). SNPs genotyped in the primary screening [3] are shown in italics. rs202573, which was genotyped both in the previous and in the present study, is underlined.

A total of 15 SNPs in a ~100-kb interval around *IL22RA2* were analyzed in the Bilbao cohort. Of these, five showed nominally significant (*p* < 0.05) association with MS (Figure 1, Table 2). The most strongly associated SNP was rs202573 (OR = 1.27, *p* = 0.007), which had already been the index SNP in the initial screening [3]. This intronic SNP is in weak LD in the Bilbao dataset (*r*^2^ = 0.06, D’ = 0.31) with the most significant GWAS-derived top SNP found at the IL22RA2 locus, i.e., rs17066096 [1,4,5,6]. Rs17066096 showed no association in the Bilbao dataset. Logistic regression analysis of all SNPs conditioning on rs202573 did not reveal any other independent SNP signal reaching statistical significance including rs17066096, suggesting that the main effect observed in this population is driven by rs202573 (Appendix A Table A1). Haplotype analysis did not show any haplotype displaying a higher significance than single SNPs in the *IL22RA2* region (Table A2).

**Figure 2:**
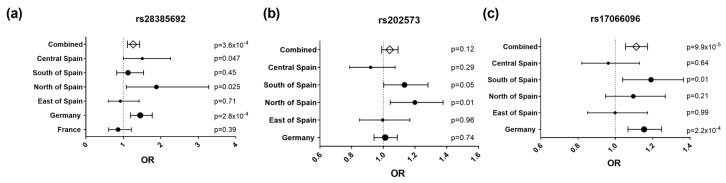
Forest plots representing effect size estimates (OR, 95% confidence interval) of the risk alleles of rs28385692 (**a**), rs202573 (**b**) and rs17066096 (**c**) in the study populations: Central Spain (Madrid area), South of Spain (Andalucía), North of Spain (Basque Country), East of Spain (Barcelona area), Germany, and France, and in the combined dataset. The dots’ size is proportional to the sample size of each population.

**Figure A1:**
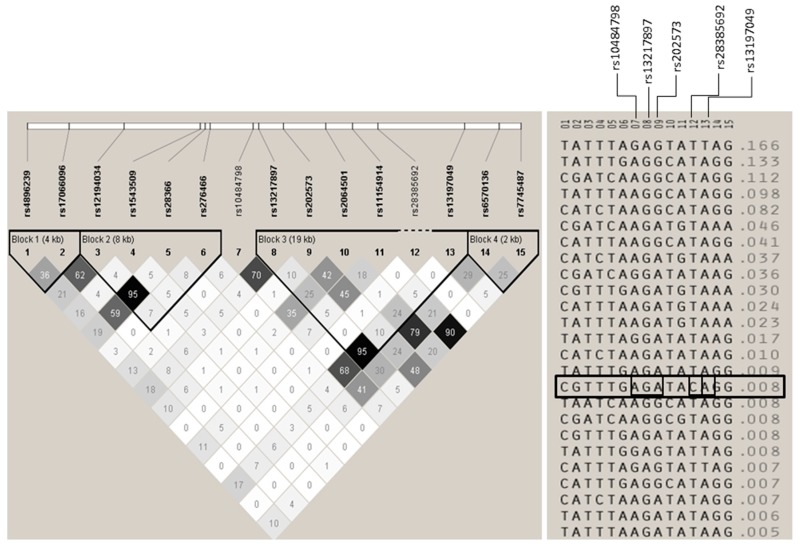
Linkage disequilibrium structure and haplotypes of *IL22RA2*. On the left, LD plot of the 15 SNPs analyzed in the Bilbao cohort. Blocks were calculated using the confidence interval algorithm implemented in Haploview [25,26]. Only samples genotyped for all SNPs were considered for the haplotype calculation. The numbers inside the squares indicate *r*^2^ values, and darker shades of gray represent higher degrees of linkage disequilibrium. On the right, all the haplotypes that were present in the Bilbao cohorts considering all SNPs in a single LD block are represented. The only haplotype containing the risk (C) allele of the non-synonymous SNP rs28385692 is boxed. SNPs with significant associations and rs28385692 are indicated on top of the right panel.

Interestingly, an infrequent non-synonymous SNP (rs28385692; changes Leu to Pro at position 16 of the SP shared by the three IL-22BP isoforms) was at the limit of statistical significance in the Bilbao dataset (Figure 1 and Table 2; *p* = 0.05, OR = 1.972, 95% CI = 0.983–3.954). The risk allele of this SNP (C) is comparatively rare (MAF = 0.015). Notably, haplotype analyses of all genotyped SNPs revealed that among all the haplotypes that were present with a frequency higher than 0.5% in the Bilbao cohort, the C allele was present only in one haplotype, which contained the risk alleles of four additional SNPs that displayed significant associations in the Bilbao dataset (Figure A1). The association of rs28385692 with MS strengthened when adding 250 controls and 572 cases from nearby Donostia to our analysis, also located in the Basque Country (*p* = 0.03, OR = 1.88, 95% CI = 1.08–3.282). Next, we validated this finding in independent Spanish and European cohorts comprising a total of 8960 cases and 7613 controls. The joint analysis of the discovery and validation cohorts confirmed the association of rs28385692 with MS (*p* = 3.6 × 10^−4^, OR = 1.262, 95% CI = 1.11–1.434 (Figure 2a).

**Figure A2:**
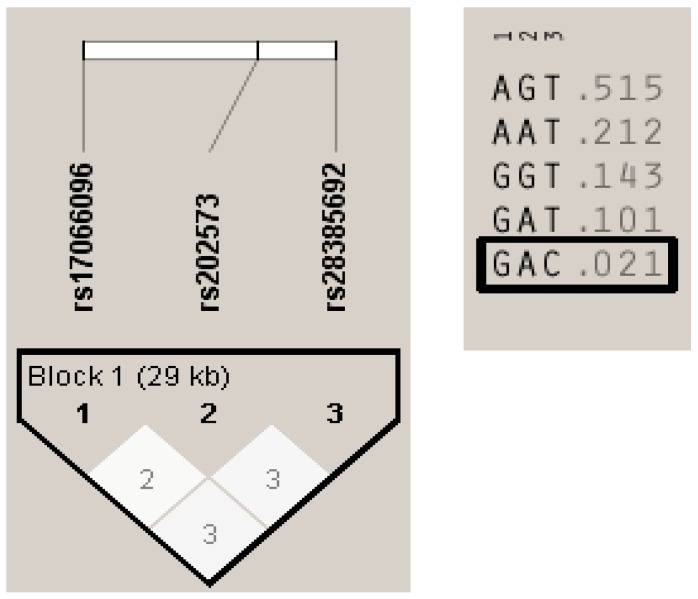
Haplotypes formed by the three SNPs genotyped in the Basque Country, Madrid, Andalucía, Barcelona and Germany populations. The three SNPs were considered in a single haplotype block. On the left, LD plot showing pairwise *r*^2^ values between the SNPs. On the right, the 5 SNP haplotypes present in the combined cohorts. The only haplotype containing the C allele of rs28385692 is boxed.

To further delineate the findings of this mapping effort, our original index SNP, rs202573, and the GWAS-derived top SNP, rs17066096 [4,6], were also genotyped in all validation datasets except France. Upon combining all available datasets, we found a significant association of rs17066096 (*p* = 9.9 × 10^−5^, OR = 1.11, 95% CI = 1.054–1.172), but not rs202573 (*p* = 0.12, OR = 1.04, 95% CI = 0.99–1.09) (Figure 2b,c). Logistic regression analysis on the combined datasets revealed that the associations of rs17066096 and rs28385692 are statistically independent, since conditioning on one SNP did not abolish association of the other (Table 3). None of the haplotypes formed by these three SNPs increased the statistical evidence for association compared to the single SNPs in the combined datasets, but, as seen in the dataset from Bilbao, the C allele of the infrequent SNP rs28385692 appeared only in the same haplotype as the risk alleles of the other two SNPs (Figure A2).

We next attempted to define which of the associated SNPs were most likely to have functional effects based on in silico predictions. To this purpose, we analyzed the five significant variants from the original Bilbao SNP screen and SNPs that were significantly associated in the validation effort, i.e., rs17066096 and rs28385692, using VEP and RegulomeDB (Table 4). As this study was based on a haplotype-tagging method, the observed association signals were considered to be representative of SNPs in linkage disequilibrium (LD) with the associated markers, and therefore, proxies for the associated SNPs were also included in the analysis. Neither rs202573, the most associated SNP in the Bilbao dataset, nor the only proxy of this SNP which met the *r*^2^ threshold (*r*^2^ = 0.8) to be included in the analysis, were predicted to overlap with any regulatory feature. rs17066096 did not show evidence to be functional either, but two of its perfect proxies (*r*^2^ = 1), rs17066063 and rs62420820, displayed a moderate regulatory potential based on RegulomeDB. rs17066063 was the SNP with the highest regulatory potential based on concordance between RegulomeDB (score 3a) and VEP (consequence: TF binding site variant). rs28385692 was predicted to lie within a regulatory region, although the level of evidence supporting this is modest (score 5 according to RegulomeDB).

### 3.2. In Silico Analysis of the p.Leu16Pro Variant

**Figure 3:**
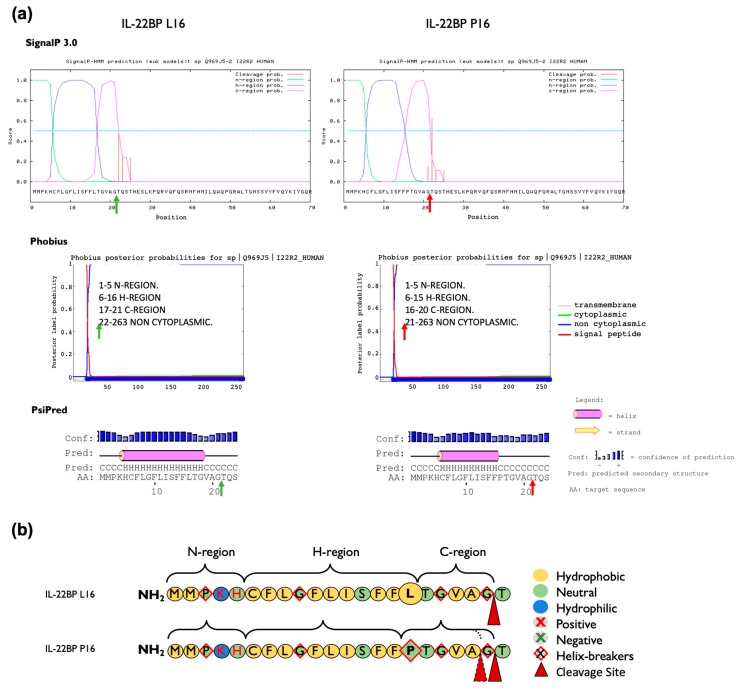
Prediction of the effect of the p.Leu16Pro amino acid change on the IL-22BP signal peptide structure and cleavage site of mature IL-22BP. (**a**) The signal peptide cleavage site indicated with green (wt, Leu16) or red (Pro16) arrows, was predicted using SignalP-3.0, Phobius and PsiPred software. The cleavage site for the canonical sequence is predicted to occur between positions 21 and 22 by the three software applications used. The p.Leu16Pro variant causes a shift in the predicted cleavage site to between position 20 and 21 according to Phobius but not SignalP 3.0 and PsiPred each of which predicted identical cleavage sites to the canonical sequence. All software coincided in predicting a decrease in the length of the H-region in the mutant form. (**b**) Representation of the composition, hydrophobicity and charge of the amino acids that comprise the signal peptide of IL-22BP. The three domain structures of the IL-22BP signal peptide are represented based on the overall results obtained in (**a**), and consist of the N-region, a hydrophilic positively charged N-terminal region; the H-region, a hydrophobic core region; and the C-region, a polar uncharged C-terminal region that is recognized by signal peptidase.

**Figure A3:**
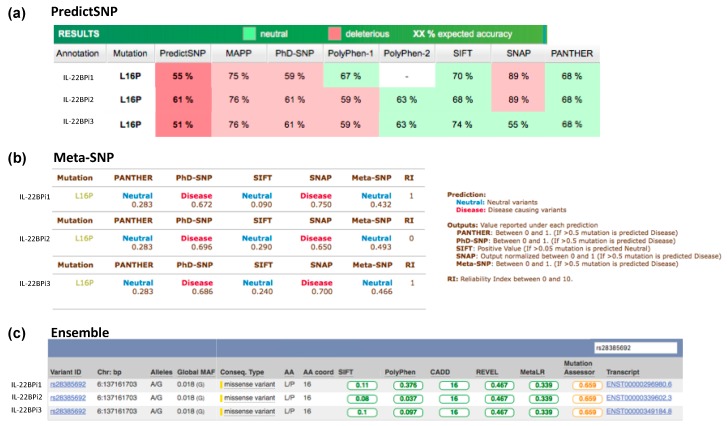
The pathogenicity prediction of rs28385692 using PredictSNP (**a**), Meta-SNP (**b**), and (**c**) Ensemble VEP web servers. The results indicate the predictive severity of the p.Leu16Pro variant conferred by the SNP rs28385692 for the three IL-22BP isoforms.

**Figure A4:**
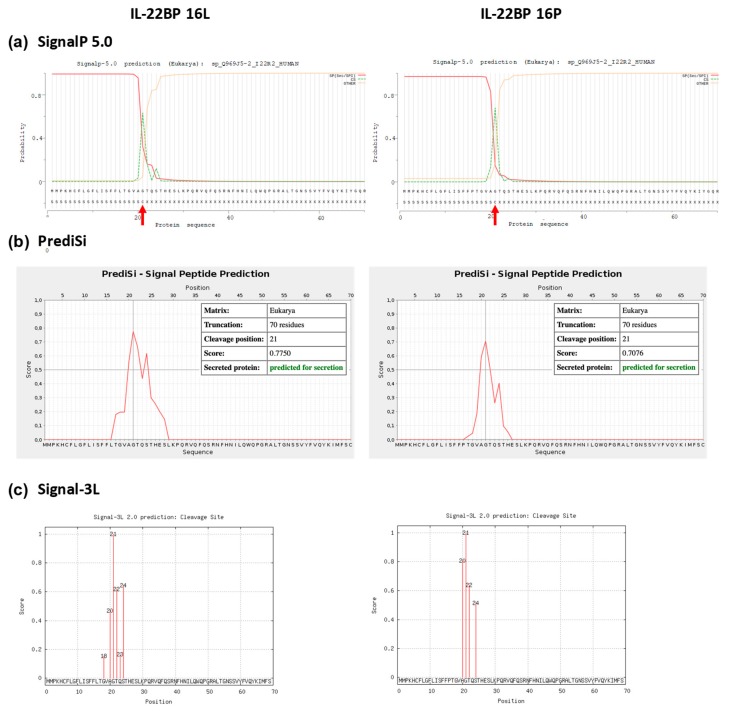

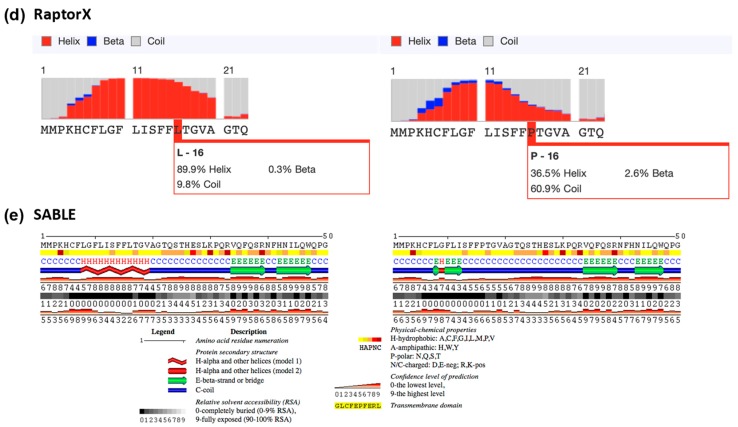
Signal peptide prediction in the Leu16 and Pro16 variant IL-22BP proteins. SignalP 5.0, PrediSi and Signal-3L predicted the cleavage site for both wild-type and p.Leu16Pro mutant to be the 21st residue of the signal peptide. Panels (**a**–**c**) show cleavage sites from SignalP 5.0, PrediSi and Signal-3L, respectively. Panels (**d**,**e**) represent the secondary structure predictions from RaptorX and SABLE, respectively.

We evaluated in silico whether rs28385692 is functionally neutral or deleterious by using computational tools based on different approaches [42]; i.e., PredictSNP [31], Meta-SNP [32] and Ensembl VEP tool [29,33]. A summary of the features of each tool used in this study is represented in Table A3. PredictSNP indicated that the overall result of the p.Leu16Pro transition in the SP was deleterious for all three isoforms (Figure A3a). However, Meta-SNP [32] anticipated a neutral effect for the p.Leu16Pro point mutation in all IL-22BP isoforms (Figure A3b). Finally, the pathogenicity prediction tools included in Ensembl VEP tool predicted the substitution not to be deleterious except for a sub-analysis based on the Mutation Assessor tool available in Ensembl VEP, which attributed a medium level of functional impact to this variant (Figure A3c). Thus, the overall results obtained with the computational tools were not robust enough to unequivocally assign a functional effect to the p.Leu16Pro variant. As this variant occurs in the SP of IL-22BP protein, its potential effect on structural aspects pertinent to SP biological function per se was assessed in more detail by in silico methods. Coding SNPs in signal peptides may alter translocation efficiency, cleavage sites, as well as post-cleavage events [43]. We analyzed the charge, the hydrophobicity, and the helix-breaker amino acid residues comprised in the SP of IL-22PB, as well as the modifications introduced by the p.Leu16Pro variant (Figure 3). The Leu16 residue is one of three leucines that contribute to the IL-22BP SP hydrophobic core H-region, a stretch of hydrophobic amino acids with propensity to form a single alpha-helix. However, its replacement, Pro16, is a helix-breaker neutral residue with restricted conformational flexibility that has no free hydrogen to contribute to helix stability. Results obtained with various signal peptide cleavage site and secondary structure prediction tools are represented in Figure 3a. While SignalP-3.0 [34] and PSIPRED [36] did not predict any change in the cleavage site, Phobius [35] predicted the p.Leu16Pro polymorphism to shift the signal peptide cleavage site from the 21st to 20th residue. The three software applications coincided in predicting a shortening of the hydrophobic core of the signal peptide. Other prediction tools were applied as well and these predicted similar structural effects (Figure A4).

### 3.3. The p.Leu16Pro Variant Decreases Secretion of IL-22BPi1, IL-22BPi2 and IL-22BPi3

**Figure 4:**
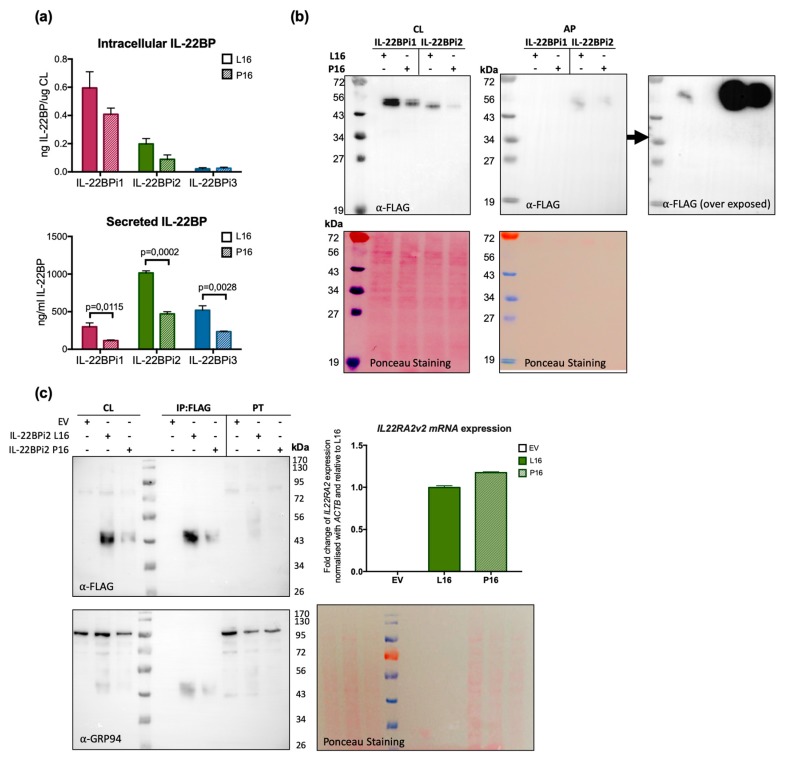
The Pro16 variant in the SP of the three IL-22BP isoforms is associated with decreased secretion levels compared to the Leu16 variant. (**a**) HEK293 cells were transfected with the indicated expression plasmids, 24 h later cells were lysed and the conditioned medium collected. Intracellular and secreted IL-22BP isoform protein levels were measured by ELISA (mean ± SEM; *n* = 3; *p*-values by unpaired *t*-test). (**b**) HEK293 cells were transfected with the indicated expression plasmids, 24 h later cells were lysed and the conditioned medium was subjected to acetone precipitation (AP). Both cell lysates (CL) and AP were resolved by SDS-PAGE under non-reducing conditions and immunoblotted against FLAG (Ponceau staining served as loading control). For AP, the immunoblot membrane was subjected to longer exposure times. (**c**) HEK293 cells were transiently transfected with the indicated expression vectors (EV denotes empty vector), 24 h later cells were lysed and RNA purified. Intracellular IL-22BP protein was immunoaffinity-purified with FLAG agaroses and detected by WB following FLAG purification and in cell lysates (CL) and pass through fraction (PT). GRP94 detection and Ponceau staining served as loading controls. Transfection efficiency was measured by IL22RA2 RT-qPCR relative to the housekeeping gene ACTB. Mean ± SEM of three technical replicates. Note that as previously observed [20], immunoreactive bands corresponding to intracellular IL-22BP isoforms appear as a series of 43 to 56 kDa bands due to differential *N*-glycosylation, with secreted IL-22BPi2 gaining ~8 kDa (56 vs. 48 kDa) due to complex *N*-glycosylation.

**Figure 5:**
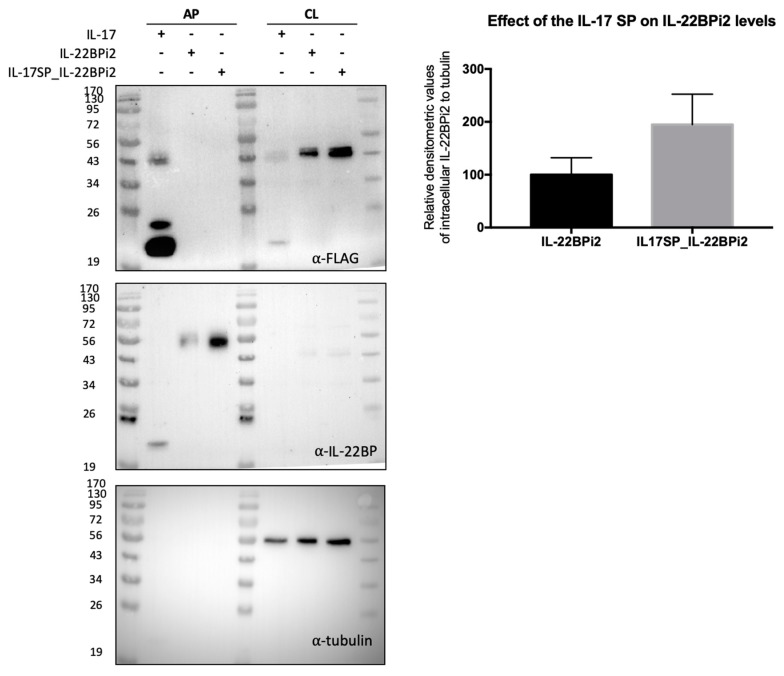
Native signal peptide of IL-22BP does not efficiently mediate secretion of IL-22BPi2. HEK293 cells were transfected with IL-17, IL-22BPi2, and IL-17SP_IL-22BPi2 expression plasmids, and 24 h later, cells were lysed and the conditioned medium subjected to acetone precipitation (AP). Both cell lysates (CL) and AP were resolved by SDS-PAGE under non-reducing conditions and immunoblotted against FLAG, IL-22BP and using tubulin as loading control. Intracellular IL-22BP reactive bands relative to tubulin ones were scanned and represented as fold change to IL-22BP wild-type (mean ± SD, *n* = 2).

**Figure A5:**
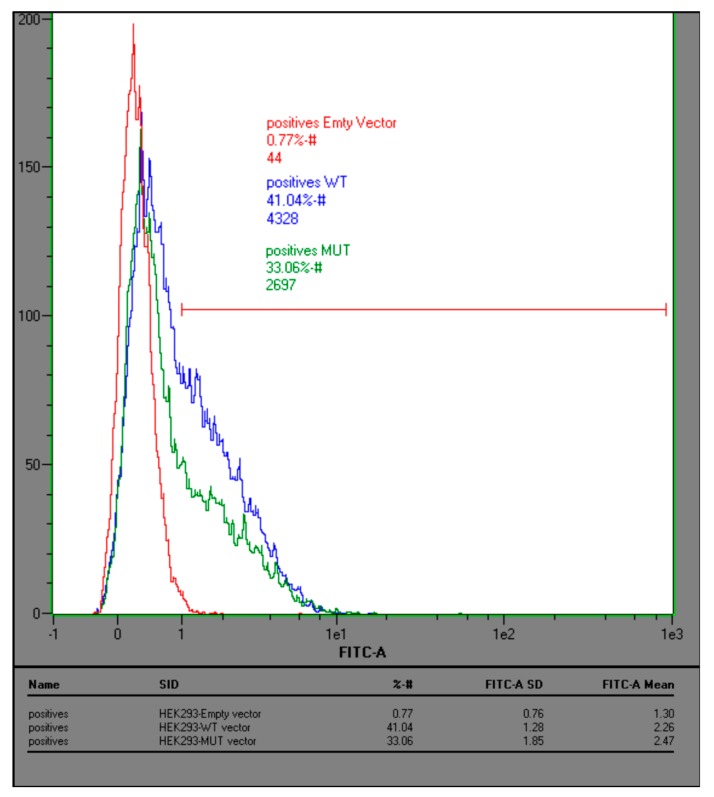
Leu16 to Pro mutation in the signal peptide of IL-22BPi2 is associated with lower intracellular levels. HEK293 cells were transfected with the *IL22RA2v2* wild type (Leu16), mutant (16P) or empty expression plasmids; 24 h later cells were fixed, permeabilized and immunostained with anti-IL22BP antibody. Cells were analyzed by flow cytometry.

To experimentally verify the effect of this variant on secretion, we performed site-directed mutagenesis to change Leu16 to Pro in the signal peptide of the three wild-type IL-22BP isoforms cloned in expression vectors reported before [20]. HEK293 cells were individually transfected with these vectors, and both intracellular and secreted levels of IL-22BPi1, IL-22BPi2 and IL-22BPi3 were measured by ELISA. Compared to Leu16, Pro16 strongly decreased the secreted levels of each isoform (Figure 4a). This was mirrored by nonsignificant trends towards decreased intracellular levels of IL-22BPi1 and IL-22BPi2. Cell lysates as well as acetone precipitates of conditioned medium of HEK293 cells transfected to produce Leu16 or Pro16 forms of IL-22BPi1 and IL-22BPi2 were analyzed by immunoblot and this revealed decreased intracellular (51 kDa for IL-22BPi1 and 48 kDa for IL-22BPi2) and secreted (56 kDa for IL-22BPi2) levels of the mutant forms (Figure 4b), while transfection efficiencies as measured by RT-qPCR through quantification of mRNA levels were similar (Figure 4c). These observations are compatible with the effects predicted by the above in silico analysis, suggesting that the Pro16 variant renders import of the precursor IL-22BP isoforms into the endoplasmic reticulum less efficient. Flow cytometry analysis also revealed a decrease of 41% to 33% in IL-22BP+ cells following transfection of HEK293 with Leu16 or Pro16 vectors encoding IL-22BPi2, respectively (Figure A5). We also assessed the efficiency of the native signal peptide of IL-22BPi2 in facilitating secretion by replacing it with that of IL-17, an efficiently secreted protein [20]. This modification resulted in much higher levels of both intracellular and secreted IL-22BPi2 (Figure 5), suggesting that the wild-type IL-22BP signal peptide is less efficiently engaged by the co-translational targeting and translocation machinery across the ER membrane than that of IL-17, which may affect overall IL-22BP biogenesis [44].

## 4. Discussion

In this study, we identified an infrequent functional variant (rs28385692) in *IL22RA2*, which confers risk to MS independently of the major GWAS-derived signal, and we were able to show that this variant has immediate functional effects by reducing secreted IL-22BP levels in transfected HEK293 cells.

SNP rs28385692, located in exon 2 of *IL22RA2*, changes the amino acid in position 16 of the protein from leucine to proline. This change occurs in the SP shared by the three IL-22BP isoforms produced by *IL22RA2*. In silico prediction methods diverged in their capacity to assign a functionally relevant effect on protein function to this variant. However, various in silico structural assessment methods coincided in predicting a shortening of the hydrophobic H-region of the IL-22BP SP by the 16P residue that may influence the precise location of the cleavage site with the mature portion of the IL-22BP proteins. Importantly, in this study, we went beyond the in silico predictions by assessing the effect of rs28385692 in vitro and showed experimentally using transfected cells that the p.Leu16Pro mutation significantly reduced the secreted levels of the IL-22BPi1, IL-22BPi2 and IL-22BPi3 isoforms. Specifically, the risk allele of p.Leu16Pro lowers secretion levels of each isoform to around 50%–60% of those of the respective wild-type forms as quantified using ELISA.

The literature documents several examples of similar changes in signal peptides that have been proven to affect secretion and are related to human diseases or traits: a Leu->Pro mutation in the signal peptide of COL5A1 (Alpha 1 Type V Collagen), a subunit of type V collagen, caused retention of this subunit in the endoplasmic reticulum and was associated with Ehlers-Danlos syndrome, a heritable connective tissue disease, in two unrelated families [45]. In addition, a common SNP causing a Leu->Pro mutation in the prohormone region of NPY (Neuropeptide Y) caused an increased secretion of this protein in chromaffin cells [46]. The inverse case, i.e., a change from proline to leucine, has also been reported to affect protein secretion: a Pro->Leu mutation in the signal peptide of DSPP (Dentin Sialophosphoprotein) resulted in a defective secretion of this protein and was associated with dentinogenesis imperfecta type III in a Korean family [47]. Finally, a Thr->Pro variant associated with coronary artery disease risk, located in the SP of the lysosomal acid lipase gene (*LIPA*), yields reduced LIPA protein levels and activity due to enhanced degradation [48].

The logistic regression analysis showed that associations of rs28385692 and the main *IL22RA2* GWAS SNP, rs17066096, with MS may be statistically independent. This suggests that there are at least two different variants causing association with MS within the *IL22RA2* locus: one presumably corresponding to rs28385692 and the other one to a SNP in LD with rs17066096. rs17066096 itself is unlikely to be a causative variant, based on its intergenic location and its missing functional effect in our extensive in silico annotations. Recently, Lill and colleagues made an attempt to discover possible causal variants in LD with rs17066096. They performed an in silico and in vitro analysis of SNPs in the 3′UTR of *IL22RA2* that were at least in moderate LD with rs17066096 (*r*^2^ > 0.3) predicted to affect the binding of micro-RNAs (miRNAs; [49]). Although they successfully identified one SNP in a miRNA binding area, they did not find allele-specific differences on this binding; therefore, association of rs17066096 with MS remains unexplained. Despite these non-confirming functional data of Lill et al., rs17066063 might be a reasonable candidate for future functional studies given its location in a regulatory region of the gene.

Our study has several limitations. Firstly, in certain sub-analyses (e.g., individual populations), our study may have been underpowered to detect modest effects. Secondly, we restricted our analyses to individuals of European descent, which was self-reported. Thus, residual confounding due to undetected population stratification may have impacted some of our results and may account for some of the heterogeneity of the observed effect estimates. However, the effect size for the GWAS-derived SNP rs17066096 estimated in our datasets (OR = 1.11) was very similar to the one described in the original GWAS (OR = 1.14) [4,5,6], suggesting that the impact of population substructure on our results is minor. Lastly, observing both genetic risk association and an effect of the genotypes on gene expression or quantitative protein production does not imply causality. Therefore, additional studies are necessary to characterize the exact molecular mechanisms of the *IL22RA2* association signal in MS.

In summary, we successfully identified a comparatively rare genetic variant in *IL22RA2* that is strongly—and likely independently of the primary GWAS signal—associated with MS. It causes an amino acid change in the signal peptide of IL-22BP and correspondingly, functional data generated for this study suggest an inhibitory effect on the trafficking of IL-22BP. Functional research on this variant will certainly provide insight about the role of this gene in MS and a better understanding of the still fairly unexplored function of IL-22BP isoforms in the immune system.

## Figures and Tables

**Table 1:** Clinical and demographic features of patients and controls included in the genetic study. ^1^ SD: standard deviation. ^2^ RR: relapsing remitting MS. ^3^ ScP: secondary progressive MS. ^4^ PP: primary progressive MS. ^5^ ND: not determined. ^6^ EDSS: expanded disability status scale.

Population		Number (% Female)	Age, Average ± SD ^1^	RR ^2^ & ScP ^3^/PP ^4^/Other/ND ^5^	Age at Onset, Average ± SD	EDSS ^6^, Mean ± SD
Bilbao	Cases	647 (72.3)	42.5 ± 12.01	79.6/9/1.4/10	30.42 ± 10.17	2.9 ± 2.3
	Controls	573 (60.3)	44.2 ± 9	-	-	-
Donostia	Cases	572 (64.8)	46.4 ± 4.8	84.8/3.8/4.8/6.6	33.01 ± 11.05	2.79 ± 2.7
	Controls	250 (66)	50.52 ± 13.26	-	-	-
Barcelona	Cases	676 (63.3)	40.17 ± 12.93	81.5/14.8/3.7	31.6 ± 9.9	3.91 ± 2.5
	Controls	910 (52.7)	40.2 ± 12.9	-	-	-
Madrid	Cases	899 (63.7)	44.8 ± 10.55	79.7/6.9/4.7/8.7	29.8 ± 8.65	2.56 ± 2.13
	Controls	697 (55.1)	40.96 ± 16.71	-	-	-
Andalucía	Cases	1474 (61)	43 ± 12	47.4/1/9/42.6	28.87 ± 10.25	ND
	Controls	1777 (64.4)	40.22 ± 12.9	-	-	-
Germany	Cases	3762 (70.2)	42.2 ± 13.6	ND	ND	ND
	Controls	2972 (60.1)	41.1 ± 14.05	-	-	-
France	Cases	1344 (63.6)	44.3 ± 11.8	ND	ND	ND
	Controls	768 (60.4)	39.6 ± 13	-	-	-

**Table 2:** Association values of SNPs included in the mapping analysis in the Bilbao dataset. ^1^ Position is according to the hg19 genome build. ^2^ RAF: risk allele frequency. ^3^ OR: odds ratio. ^4^ CI: confidence interval.

SNP	Position ^1^	Risk Allele	RAF ^2^ Cases	RAF Controls	Other Allele	*p*	OR ^3^ (95% CI ^4^)
rs4896239	137,448,873	C	0.52	0.50	T	0.19	1.116 (0.942–1.31)
rs17066096	137,452,908	G	0.29	0.27	A	0.26	1.132 (0.92–1.34)
rs12194034	137,458,262	A	0.23	0.22	T	0.65	1.047 (0.86–1.274)
rs1543509	137,465,656	C	0.15	0.14	T	0.92	1.012 (0.797–1.285)
rs28366	137,466,087	C	0.24	0.23	T	0.52	1.066 (0.88–1.297)
rs276466	137,466,614	A	0.78	0.78	G	0.99	1.001 (0.799–1.25)
rs10484798	137,470,756	A	0.76	0.72	G	0.05	1.23 (1.0–1.508)
rs13217897	137,471,327	G	0.83	0.79	A	0.02	1.291 (1.05–1.591)
rs202573	137,473,672	A	0.33	0.28	G	0.007	1.273 (1.067–1.518)
rs2064501	137,477,823	T	0.50	0.49	C	0.65	1.039 (0.879–1.226)
rs11154914	137,480,411	G	0.19	0.16	A	0.06	1.23 (0.99–1.524)
rs28385692	137,482,840	C	0.02	0.01	T	0.05	1.972 (0.983–3.954)
rs13197049	137,491,211	A	0.83	0.80	T	0.03	1.260 (1.021–1.556)
rs6570136	137,494,622	A	0.46	0.45	G	0.85	1.017 (0.847–1.222)
rs7745487	137,496,672	A	0.18	0.15	G	0.10	1.201 (0.96–1.496)

**Table 3:** Association values of the three SNPs in the discovery + validation datasets conditioned on rs17066096 and rs28385692. ^1^ OD: odds ratio. ^2^ CI: confidence interval.

		Conditioned to rs17066096		Conditioned to rs28385692	
SNP	Reference (minor) Allele	*p*	OR ^1^ (95% CI ^2^)	*p*	OR (95% CI)
rs17066096	G	NA	NA	0.001042	1.098 (1.039–1.162)
rs202573	A	0.2424	1.029 (0.981–1.079)	0.3093	1.033 (0.9702–1.1)
rs28385692	C	0.001146	1.098 (1.101–1.476)	NA	NA

**Table 4:** Functional predictions of associated SNPs and proxies. SNPs with significant associations in the mapping exercise or in the discovery + validation cohorts and their proxies (*r*^2^ > 0.8 in 1000 Genomes Phase III CEU population) were assessed using VEP and RegulomeDB. ^1^ Minor allele frequency is based on European populations in the 1000 Genomes Project Phase III.

SNP	Proxy	Major Allele	Minor Allele (Frequency) ^1^	Ensembl Consequence	SIFT	PolyPhen	RegulomeDB
rs10484798	rs28362847	G	A (0.21)	regulatory_region_variant	-	-	5: TF binding or DNase peak
	rs10484798	G	A (0.21)	intron_variant	-	-	6: other
rs13197049	rs13217897	G	A (0.17)	intron_variant	-	-	3a: TF binding + any motif + DNase peak
	rs17175239	A	G (0.17)	intergenic_variant	-	-	5: TF binding or DNase peak
	rs1961618	C	T (0.17)	intron_variant	-	-	5: TF binding or DNase peak
	rs12664889	C	A (0.17)	intron_variant	-	-	7: no data
	rs13197049	A	T (0.17)	intron variant	-	-	7: no data
	rs11154913	A	G (0.17)	intron_variant	-	-	5: TF binding or DNase peak
	rs13193435	C	A (0.17)	intron_variant	-	-	5: TF binding or DNase peak
	rs7749054	T	G (0.17)	intergenic_variant	-	-	6: other
	rs13197049	A	T (0.17)	intron_variant	-	-	7: no data
	rs7766677	A	C (0.17)	intergenic_variant	-	-	7: no data
rs13217897	rs13217897	G	A (0.17)	intron_variant	-	-	3a: TF binding + any motif + DNase peak
	rs13193435	C	A (0.17)	intron variant	-	-	5: TF binding or DNase peak
	rs1961618	C	T (0.17)	intron_variant	-	-	5: TF binding or DNase peak
	rs17175239	A	G (0.17)	intergenic_variant	-	-	5: TF binding or DNase peak
	rs7766677	A	C (0.17)	intergenic_variant	-	-	6: other
	rs11154913	A	G (0.17)	intron_variant	-	-	7: no data
	rs7749054	T	G (0.17)	intergenic_variant	-	-	7: no data
	rs12664889	C	A (0.17)	intron_variant	-	-	7: no data
	rs13197049	A	T (0.17)	intron_variant	-	-	7: no data
rs17066096	rs17066063	G	A (0.23)	TF_binding_site_variant	-	-	3a: TF binding + any motif + DNase peak
	rs62420820	G	A (0.23)	regulatory_region_variant	-	-	3a: TF binding + any motif + DNase peak
	rs72975618	C	T (0.23)	TF_binding_site_variant	-	-	4: TF binding + DNase peak
	rs1322553	A	G (0.23)	regulatory_region_variant	-	-	5: TF binding or DNase peak
	rs12214115	G	T (0.23)	regulatory_region_variant	-	-	5: TF binding or DNase peak
	rs12214014	C	T (0.23)	regulatory_region_variant	-	-	5: TF binding or DNase peak
	rs17066096	A	G (0.23)	intergenic_variant	-	-	6: other
rs202573	rs202571	T	C (0.31)	intron_variant	-	-	7: no data
	rs202573	G	A (0.31)	intron_variant	-	-	7: no data
rs28385692	rs28385692	T	C (0.03)	missense_variant	Tolerated (0.11)	Benign (0.376)	5: TF binding or DNase peak

## Abbreviations

AP	Acetone precipitate
CL	Cell lysate
EAE	Experimental autoimmune encephalomyelitis
EDSS	Expanded Disability Status Scale
GWAS	Genome-wide association screen
IL-22BPi1, 2 or 3	Interleukin 22 binding protein isoform-1, -2 or -3
*IL22RA2*	Interleukin 22 receptor subunit alpha 2
LD	Linkage disequilibrium
MAF	Minor allele frequency
OR	Odds ratio
PP	Primary progressive
RAF	Risk allele frequency
RR	Relapsing-remitting
ScP	Secondary progressive
SFM	Serum-free medium
SNP	Single nucleotide polymorphism
SP	Signal peptide

## Appendix A

**Table A1:** Association values of all SNPs included in the fine mapping conditioned to the index SNP, rs202573. ^1^ OR: odds ratio. ^2^ CI: confidence interval.

SNP	OR ^1^ (95% CI ^2^)	*p*
rs4896239	0.95 (0.798–1.131)	0.5638
rs17066096	1.059 (0.8746–1.282)	0.5569
rs12194034	1.025 (0.8412–1.249)	0.8055
rs1543509	0.9821 (0.7719–1.25)	0.8835
rs28366	1.047 (0.8594–1.275)	0.6497
rs276466	0.9779 (0.7803–1.225)	0.8458
rs10484798	0.8452 (0.6874–1.039)	01105
rs13217897	0.8249 (0.6608–1.03)	0.08906
rs2064501	0.8337 (0.6674–1.041)	0.109
rs11154914	1.043 (0.7864–1.384)	0.7687
rs28385692	1.799 (0.8654–3.739)	0.1158
rs13197049	0.846 (0.6766–1.058)	0.1423
rs6570136	0.8669 (0.7018–1.071)	0.1855
rs7745487	1.001 (0.7436–1.347)	0.9947

**Table A2:** Association values of the haplotypes in blocks. LD blocks were calculated with the confidence interval method using Haploview [25,26].

Block	Haplotype	Frequency	Case, Control Frequencies	*p*
rs4896239 + rs28385692	TA	0.501	0.479, 0.519	0.102
	CG	0.267	0.273, 0.262	0.6031
	CA	0.232	0.248, 0.219	0.1642
rs12194034 + rs1543509+rs28366 + rs276466	TTTA	0.408	0.404, 0.411	0.763
	TTTG	0.22	0.216, 0.224	0.7026
	ATCA	0.217	0.215, 0.218	0.8637
	TCTA	0.148	0.160, 0.139	0.2266
rs132117897 + rs202573 + rs2064501 + rs11154914 + rs13197049	GGCAA	0.495	0.475, 0.512	0.1298
	AGTAT	0.193	0.178, 0.206	0.1565
	GATGA	0.172	0.192, 0.156	0.0561
	GATAA	0.124	0.139, 0.111	0.0806
rs6570136 +rs7745487	GG	0.551	0.543, 0.558	0.5396
	AG	0.278	0.265, 0.289	0.2853
	AA	0.171	0.192, 0.153	0.0373

**Table A3:** Features of the individual and consensus computational tools used in this study. Adapted from [42].

ID Server Name	Individual or Consensus Tool	Website	MLT That Based on	Input Parameters	Deleterious Threshold & Outputs	Ref.
**Meta RL & Meta SVM**	Consensus	http://annovar.openbioinformatics.org/en/latest/	MMAF, linear kernel, radial kernel and polynomial kernel	A score of SIFT, PolyPhen-2, GERPþþ, Mutation Taster, Mutation Assessor, FATHMM, LRT, SiPhy and PhyloP	D (Deleterious), N (Neutral) and U (Unknown)	[50]
**Meta-SNP**	Consensus	http://snps.biofold.org/meta-snp/	RF	A score of PANTHER, PhD-SNP, SIFT, and SNAP	Disease related or Polymorphic non-synonymous SNVs	[32]
**PredictSNP**	Consensus	https://loschmidt.chemi.muni.cz/predictsnp/	Weighted majority vote consensus	A score of PolyPhen-1, PolyPhen-2, SIFT, MAPP, PhD-SNP and SNAP	Confidence scores and neutral or deleterious	[31]
**REVEL**	Consensus	https://omictools.com/revel-tool	RF	A score of MutPred, FATHMM, VEST, Poly-Phen, SIFT, PROVEAN, Mutation Assessor, Mutation Taster, LRT, GERP, SiPhy, phyloP, and phastCons	Disease variants or rare neutral variants	[51]
**CADD**	Individual	http://cadd.gs.washington.edu/score	A linear kernel support vector machine (SVM)	SNVs	Functional, deleterious, and pathogenic variants	[52]
**MAPP**	Individual	http://mendel.stanford.edu/SidowLab/downloads/MAPP/index.html	Physicochemical properties and alignment score	The original amino acid, the position of the substitution and the new amino acid.	Score (0–1). The predicted is damaging if the score <=0.05 and tolerated if the score >0.05	[53]
**Mutation Assessor**	Individual	http://mutationassessor.org/r3/	Conservation method	Genome build, chromosome position, reference allele and substituted allele or Protein ID and variant	(VC) Variant conservation score and (VS) Variant specificity score. Level of functional impact (high, medium, low, neutral)	[54]
**SIFT**	Individual	http://sift.jcvi.org/www/SIFT_help.html#SIFT_OUTPUT	Alignment scores	The original amino acid, the position of the substitution and the new amino acid	Score (0–1). The predicted is damaging if the score <=0.05 and tolerated if the score >0.05	[55]
**SNAP2**	Individual	https://rostlab.org/services/snap2web/	ANN	Protein sequence	Non-neutral and neutral, Score and accuracy	[56]
**PANTHER**	Individual	http://www.pantherdb.org/about.jsp	Alignment Scores HMM	Protein sequence and substitution	All GO annotations & Phylogenetic annotation	[57]
**PhD-SNPg**	Individual	http://snps.biofold.org/phd-snpg/method.html	Gradient boosting algorithm	Chromosome position, and protein variation (position, and first amino acid, and second amino acid variant)	If the probability is >0.5 then the SNV is predicted to be Pathogenic otherwise Benign	[58]
**Polyphen-2**	Individual	http://genetics.bwh.harvard.edu/pph2/	Empirical rules	Protein, SNP identifier or Protein sequence in FASTA format and positions of the substitution	Probably damaging or Benign, or Possibly damaging, Sensitivity, specificity, Multiple sequence alignment and 3D Visualization	[59]
